# 1-(4-Cyano­benz­yl)-4-methyl­pyridinium bromide

**DOI:** 10.1107/S1600536809020054

**Published:** 2009-06-06

**Authors:** Hong Chen, Kun Zhu, Guang-Xiang Liu

**Affiliations:** a Anhui Key Laboratory of Functional Coordination Compounds, School of Chemistry and Chemical Engineering, Anqing Normal University, Anqing 246003, People’s Republic of China

## Abstract

In the title compound, C_14_H_13_N_2_^+^·Br^−^, the 1-(4-cyano­benz­yl)-4-methyl­pyridinium cation has a Λ-shaped conformation, and the dihedral angle between the benzene and pyridinium rings is 75.8 (2)°. In the crystal, two cations form a dimer through π–π inter­actions between pyridine rings [the centroid–centroid distance is 3.685 (1) Å].

## Related literature

For cations with similar geometry, see: Liu *et al.* (2007 ▶, 2008 ▶).
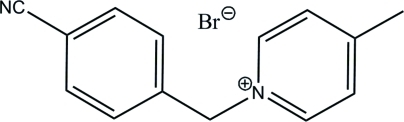


## Experimental

### 

#### Crystal data

C_14_H_13_N_2_^+^·Br^−^
*M* *_r_* = 289.17
Monoclinic, 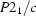

*a* = 12.967 (5) Å
*b* = 8.217 (4) Å
*c* = 12.260 (5) Å
β = 96.900 (5)°
*V* = 1296.8 (10) Å^3^
*Z* = 4
Mo *K*α radiation
μ = 3.15 mm^−1^
*T* = 296 K
0.24 × 0.20 × 0.16 mm

#### Data collection

Bruker SMART APEX CCD area-detector diffractometer
Absorption correction: multi-scan (*SADABS*; Bruker, 2000 ▶) *T*_min_ = 0.493, *T*_max_ = 0.601
6270 measured reflections
2298 independent reflections
1945 reflections with *I* > 2σ(*I*)
*R*_int_ = 0.106

#### Refinement

*R*[*F* ^2^ > 2σ(*F* ^2^)] = 0.066
*wR*(*F* ^2^) = 0.204
*S* = 1.04
2298 reflections
155 parameters
H-atom parameters constrained
Δρ_max_ = 0.84 e Å^−3^
Δρ_min_ = −0.82 e Å^−3^

### 

Data collection: *SMART* (Bruker, 2000 ▶); cell refinement: *SAINT* (Bruker, 2000 ▶); data reduction: *SAINT*; program(s) used to solve structure: *SHELXS97* (Sheldrick, 2008 ▶); program(s) used to refine structure: *SHELXL97* (Sheldrick, 2008 ▶); molecular graphics: *SHELXTL* (Sheldrick, 2008 ▶); software used to prepare material for publication: *SHELXTL*.

## Supplementary Material

Crystal structure: contains datablocks I, global. DOI: 10.1107/S1600536809020054/bq2140sup1.cif

Structure factors: contains datablocks I. DOI: 10.1107/S1600536809020054/bq2140Isup2.hkl

Additional supplementary materials:  crystallographic information; 3D view; checkCIF report

## Figures and Tables

**Fig. 1.**
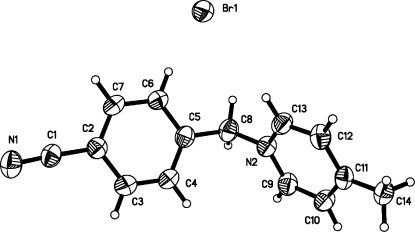
The asymmetric unit of the title compound. Displacement ellipsoids are drawn at the 30% probability level.

**Fig. 2.**
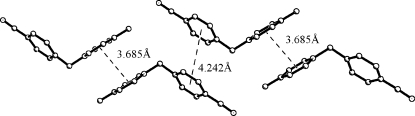
One dimensional chain is formed π–π interaction along the a-axis.

## supplementary crystallographic information

### Comment

The asymmetric unit of (I) contains one cation and one Br anion (Fig. 1). The cation has a Λ-shaped conformation, and the dihedral angles formed by the C5/C8/N2 plane with the benzene and pyridinium rings are 61.19 (2)° and 72.88 (2)°, respectively (75.8 (2)° between the benzene and pyridinium rings). The geometry of the cation is similar to the one observed in Liu *et al.* (2008) and Liu *et al.* (2007). Two cations form a dimer through π–π interaction between pyridine rings, the distance of centroid-to-centroid is 3.685Å, which further are linked into one dimensional chain by the π–π interaction between benzene ring, the distance of centroid-to-centroid is 4.242Å. A three-dimensional supramolecular structure was packed *via* Van der Waals forces (Fig. 2).

### Experimental

4-cyanobenzyl bromide (10 mmol, 1.96 g) and 4-methylpyridine (20 mmol, 1.88 g) were added to 40 ml of acetone. After stirring and refluxing for 12 h, the mixture was filtered, and the clear solution was allowed to evaporate slowly under inert atmosphere. Block crystals of the title compound were obtained after 3 days. The crystals were filtered, washed by acetone and dried in air.

### Refinement

H atoms were positioned geometrically, with C—H = 0.93Å, 0.96Å and 0.97Å for aromatic, methylene and methyl H atoms, respectively, and constrained to ride on their parent atoms, with *U*_iso_(H) = xUeq(C), where *x* = 1.5 for methyl H and *x* = 1.2 for other H atoms. The deepest hole is located 1.12Å from atom C16.

### Crystal data

**Table:**

C_14_H_13_N_2_^+^·Br^−^	*F*(000) = 584
*M**_r_* = 289.17	*D*_x_ = 1.481 Mg m^−^^3^
Monoclinic, *P*2_1_/*c*	Mo *K*α radiation, λ = 0.71073 Å
Hall symbol: -P 2ybc	Cell parameters from 3626 reflections
*a* = 12.967 (5) Å	θ = 2.9–27.7°
*b* = 8.217 (4) Å	µ = 3.15 mm^−^^1^
*c* = 12.260 (5) Å	*T* = 296 K
β = 96.900 (5)°	Block, colorless
*V* = 1296.8 (10) Å^3^	0.24 × 0.20 × 0.16 mm
*Z* = 4	

### Data collection

**Table:**

Bruker SMART APEX CCD area-detector diffractometer	2298 independent reflections
Radiation source: sealed tube	1945 reflections with *I* > 2σ(*I*)
graphite	*R*_int_ = 0.106
φ and ω scans	θ_max_ = 25.1°, θ_min_ = 1.6°
Absorption correction: multi-scan (*SADABS*; Bruker, 2000)	*h* = −15→15
*T*_min_ = 0.493, *T*_max_ = 0.601	*k* = −9→8
6270 measured reflections	*l* = −13→14

### Refinement

**Table:**

Refinement on *F*^2^	Primary atom site location: structure-invariant direct methods
Least-squares matrix: full	Secondary atom site location: difference Fourier map
*R*[*F*^2^ > 2σ(*F*^2^)] = 0.066	Hydrogen site location: inferred from neighbouring sites
*wR*(*F*^2^) = 0.204	H-atom parameters constrained
*S* = 1.04	*w* = 1/[σ^2^(*F*_o_^2^) + (0.1306*P*)^2^ + 1.6412*P*] where *P* = (*F*_o_^2^ + 2*F*_c_^2^)/3
2298 reflections	(Δ/σ)_max_ < 0.001
155 parameters	Δρ_max_ = 0.84 e Å^−^^3^
0 restraints	Δρ_min_ = −0.82 e Å^−^^3^

### Special details

**Table:**

Geometry. All e.s.d.'s (except the e.s.d. in the dihedral angle between two l.s. planes) are estimated using the full covariance matrix. The cell e.s.d.'s are taken into account individually in the estimation of e.s.d.'s in distances, angles and torsion angles; correlations between e.s.d.'s in cell parameters are only used when they are defined by crystal symmetry. An approximate (isotropic) treatment of cell e.s.d.'s is used for estimating e.s.d.'s involving l.s. planes.
Refinement. Refinement of *F*^2^ against ALL reflections. The weighted *R*-factor *wR* and goodness of fit *S* are based on *F*^2^, conventional *R*-factors *R* are based on *F*, with *F* set to zero for negative *F*^2^. The threshold expression of *F*^2^ > σ(*F*^2^) is used only for calculating *R*-factors(gt) *etc*. and is not relevant to the choice of reflections for refinement. *R*-factors based on *F*^2^ are statistically about twice as large as those based on *F*, and *R*- factors based on ALL data will be even larger.

### Fractional atomic coordinates and isotropic or equivalent isotropic displacement parameters (Å^2^)

**Table:**

	*x*	*y*	*z*	*U*_iso_*/*U*_eq_	
Br1	0.77449 (4)	0.08544 (6)	0.80230 (4)	0.0499 (3)	
C1	0.3632 (6)	0.5777 (8)	0.3983 (7)	0.0690 (18)	
C2	0.4519 (5)	0.6742 (8)	0.4207 (5)	0.0618 (15)	
C3	0.5131 (6)	0.7037 (11)	0.3372 (6)	0.084 (2)	
H3	0.4949	0.6579	0.2682	0.101*	
C4	0.6000 (5)	0.7993 (10)	0.3554 (6)	0.0783 (19)	
H4	0.6401	0.8173	0.2986	0.094*	
C5	0.6291 (5)	0.8699 (8)	0.4575 (6)	0.0639 (15)	
C6	0.5696 (5)	0.8356 (9)	0.5418 (5)	0.0664 (16)	
H6	0.5889	0.8784	0.6115	0.080*	
C7	0.4814 (5)	0.7378 (8)	0.5231 (6)	0.0672 (16)	
H7	0.4425	0.7156	0.5803	0.081*	
C8	0.7202 (5)	0.9829 (9)	0.4770 (6)	0.0728 (17)	
H8A	0.7083	1.0755	0.4282	0.087*	
H8B	0.7251	1.0231	0.5518	0.087*	
C9	0.8667 (5)	0.9449 (9)	0.3689 (7)	0.0723 (18)	
H9	0.8355	1.0195	0.3182	0.087*	
C10	0.9583 (6)	0.8757 (9)	0.3518 (6)	0.0716 (17)	
H10	0.9882	0.9006	0.2886	0.086*	
C11	1.0081 (5)	0.7671 (7)	0.4288 (6)	0.0644 (16)	
C12	0.9567 (5)	0.7273 (8)	0.5214 (7)	0.0741 (19)	
H12	0.9871	0.6548	0.5740	0.089*	
C13	0.8650 (5)	0.7938 (9)	0.5326 (6)	0.0719 (17)	
H13	0.8308	0.7629	0.5918	0.086*	
N1	0.2896 (5)	0.4920 (10)	0.3754 (6)	0.0880 (18)	
N2	0.8195 (5)	0.9065 (5)	0.4596 (5)	0.0624 (14)	
C14	1.0990 (5)	0.6989 (8)	0.4164 (6)	0.0692 (17)	
H14A	1.1067	0.5998	0.4581	0.104*	
H14B	1.1542	0.7724	0.4422	0.104*	
H14C	1.1018	0.6753	0.3402	0.104*	

### Atomic displacement parameters (Å^2^)

**Table:**

	*U*^11^	*U*^22^	*U*^33^	*U*^12^	*U*^13^	*U*^23^
Br1	0.0502 (4)	0.0532 (4)	0.0468 (4)	−0.00751 (19)	0.0074 (3)	−0.00020 (19)
C1	0.056 (4)	0.074 (4)	0.078 (5)	0.005 (3)	0.014 (3)	0.005 (3)
C2	0.057 (3)	0.063 (4)	0.065 (4)	0.009 (3)	0.006 (3)	0.007 (3)
C3	0.083 (5)	0.107 (6)	0.065 (4)	−0.016 (4)	0.019 (4)	−0.004 (4)
C4	0.070 (4)	0.105 (5)	0.064 (4)	−0.013 (4)	0.022 (3)	−0.003 (4)
C5	0.058 (3)	0.060 (3)	0.075 (4)	0.007 (3)	0.012 (3)	0.003 (3)
C6	0.066 (4)	0.075 (4)	0.060 (4)	−0.003 (3)	0.016 (3)	−0.006 (3)
C7	0.067 (4)	0.074 (4)	0.064 (4)	0.003 (3)	0.025 (3)	0.006 (3)
C8	0.062 (4)	0.069 (4)	0.088 (5)	0.005 (3)	0.016 (3)	−0.002 (4)
C9	0.061 (4)	0.078 (4)	0.078 (5)	−0.002 (3)	0.008 (3)	0.012 (3)
C10	0.066 (4)	0.079 (4)	0.073 (4)	0.003 (3)	0.020 (3)	0.003 (4)
C11	0.051 (3)	0.062 (4)	0.081 (4)	−0.006 (3)	0.010 (3)	−0.002 (3)
C12	0.061 (4)	0.073 (4)	0.088 (5)	0.001 (3)	0.003 (3)	0.010 (4)
C13	0.066 (4)	0.082 (4)	0.071 (4)	−0.002 (3)	0.020 (3)	0.008 (3)
N1	0.062 (3)	0.095 (5)	0.106 (5)	−0.002 (4)	0.011 (3)	−0.002 (4)
N2	0.059 (3)	0.063 (3)	0.066 (4)	0.002 (2)	0.012 (3)	0.006 (2)
C14	0.055 (3)	0.068 (4)	0.086 (5)	0.002 (3)	0.014 (3)	−0.005 (3)

### Geometric parameters (Å, °)

**Table:**

C1—N1	1.192 (9)	C8—H8B	0.9700
C1—C2	1.397 (10)	C9—C10	1.355 (10)
C2—C7	1.371 (9)	C9—N2	1.370 (10)
C2—C3	1.390 (10)	C9—H9	0.9300
C3—C4	1.370 (11)	C10—C11	1.399 (10)
C3—H3	0.9300	C10—H10	0.9300
C4—C5	1.389 (10)	C11—C14	1.330 (9)
C4—H4	0.9300	C11—C12	1.423 (10)
C5—C6	1.392 (9)	C12—C13	1.330 (10)
C5—C8	1.499 (9)	C12—H12	0.9300
C6—C7	1.394 (9)	C13—N2	1.371 (9)
C6—H6	0.9300	C13—H13	0.9300
C7—H7	0.9300	C14—H14A	0.9600
C8—N2	1.471 (8)	C14—H14B	0.9600
C8—H8A	0.9700	C14—H14C	0.9600
			
N1—C1—C2	177.0 (8)	C10—C9—N2	121.0 (7)
C7—C2—C3	119.1 (6)	C10—C9—H9	119.5
C7—C2—C1	122.0 (6)	N2—C9—H9	119.5
C3—C2—C1	118.9 (7)	C9—C10—C11	120.4 (7)
C4—C3—C2	120.7 (7)	C9—C10—H10	119.8
C4—C3—H3	119.6	C11—C10—H10	119.8
C2—C3—H3	119.6	C14—C11—C10	122.4 (7)
C3—C4—C5	121.0 (6)	C14—C11—C12	120.1 (7)
C3—C4—H4	119.5	C10—C11—C12	117.5 (6)
C5—C4—H4	119.5	C13—C12—C11	119.9 (7)
C4—C5—C6	118.0 (6)	C13—C12—H12	120.0
C4—C5—C8	121.8 (6)	C11—C12—H12	120.0
C6—C5—C8	120.2 (6)	C12—C13—N2	122.0 (7)
C5—C6—C7	120.8 (6)	C12—C13—H13	119.0
C5—C6—H6	119.6	N2—C13—H13	119.0
C7—C6—H6	119.6	C9—N2—C13	119.1 (6)
C2—C7—C6	120.3 (6)	C9—N2—C8	120.3 (6)
C2—C7—H7	119.9	C13—N2—C8	120.6 (6)
C6—C7—H7	119.9	C11—C14—H14A	109.5
N2—C8—C5	113.5 (6)	C11—C14—H14B	109.5
N2—C8—H8A	108.9	H14A—C14—H14B	109.5
C5—C8—H8A	108.9	C11—C14—H14C	109.5
N2—C8—H8B	108.9	H14A—C14—H14C	109.5
C5—C8—H8B	108.9	H14B—C14—H14C	109.5
H8A—C8—H8B	107.7		
			
C7—C2—C3—C4	−2.2 (12)	N2—C9—C10—C11	−2.0 (11)
C1—C2—C3—C4	179.2 (7)	C9—C10—C11—C14	−178.6 (7)
C2—C3—C4—C5	−0.2 (13)	C9—C10—C11—C12	2.9 (11)
C3—C4—C5—C6	2.4 (11)	C14—C11—C12—C13	−179.0 (7)
C3—C4—C5—C8	−176.3 (7)	C10—C11—C12—C13	−0.5 (11)
C4—C5—C6—C7	−2.1 (10)	C11—C12—C13—N2	−2.9 (11)
C8—C5—C6—C7	176.5 (6)	C10—C9—N2—C13	−1.4 (10)
C3—C2—C7—C6	2.4 (10)	C10—C9—N2—C8	179.9 (7)
C1—C2—C7—C6	−179.0 (6)	C12—C13—N2—C9	3.9 (11)
C5—C6—C7—C2	−0.2 (10)	C12—C13—N2—C8	−177.4 (7)
C4—C5—C8—N2	−61.6 (9)	C5—C8—N2—C9	107.0 (7)
C6—C5—C8—N2	119.8 (7)	C5—C8—N2—C13	−71.7 (8)
